# Development of hexaplex PCR assay for the detection and characterization common tick-borne pathogens in dogs, and analysis of risk factors

**DOI:** 10.1016/j.vas.2026.100705

**Published:** 2026-05-19

**Authors:** Mirashree Pati, Ramesh Chandra Patra, Manaswini Dehuri, Smruti Ranjan Mishra, Sangram Biswal, Geeta Rani Jena, Chinmoy Mishra, Dillip Kumar Karna

**Affiliations:** a Department of Veterinary Medicine, College of Veterinary Science and Animal Husbandry, Odisha University of Agriculture and Technology, Bhubaneswar, Pin-751003, India; b Department of Veterinary Parasitology, College of Veterinary Science and Animal Husbandry, Odisha University of Agriculture and Technology, Bhubaneswar, Pin-751003, India; c Department of Veterinary Physiology, College of Veterinary Science and Animal Husbandry, Odisha University of Agriculture and Technology, Bhubaneswar, Pin-751003, India; d Department of Animal Breeding and Genetics, College of Veterinary Science and Animal Husbandry, Odisha University of Agriculture and Technology, Bhubaneswar, Pin-751003, India

**Keywords:** Tick-borne hemoparasite, mPCR, Risk factor, Sensitivity, Epidemiology

## Abstract

The present investigation was intended to develop and validate an internally controlled hexaplex PCR assay for concurrent detection and differentiation of the five major canine tick-borne pathogens (TBP) namely, *Ehrlichia canis, Babesia gibsoni, Babesia vogeli, Hepatozoon canis* and *Anaplasma platy*s in 8 selected districts of Odisha, India. The assay generated amplicons of 817 bp, 737 bp, 602 bp, 489 bp and 292 bp corresponding to *E. canis* (16S rRNA gene)*, B. vogeli, B. gibsoni, H. canis* (18S rRNA gene) and *A. platy*s (HSP GroEL gene), along with amplicon of 218 bp targeting canine β-actin gene as internal amplification control (IAC) to avoid false negative result. The kappa values for diagnostic specificity and sensitivity of multiplex PCR assay in detection of the pathogens revealed very good agreement with their singleplex PCR counterparts (n = 61). The analytical sensitivity of multiplex PCR assay was recorded to be 0.1 pg for detection of *A. platys, H. canis* and *B. vogeli,* and 10 pg for *E. canis* and *B. gibsoni*. The epidemiological survey (n = 320) revealed 31.56% of TBP infection and incidence of *H. canis, B. gibsoni* were high in summer season and *A. platys* in rainy. Co-infections exacerbated clinical affections. The generated sequence of the Odisha isolates of these pathogens in this study revealed 98–100% homology with various global isolates of these pathogens. The standardized multiplex PCR developed in the present study, can be used as a rapid, economical and sensitive diagnostic tool for identifying tick-borne haemoparasites in dogs.

## Highlights

• The developed hexaplex PCR assay using internal amplification control (IAC) successfully detected the five major canine tick-borne hemoparasites.
• The analytical sensitivity of multiplex PCR assay was recorded to be 0.1 pg for detection of A. platys, H. canis and B. vogeli, and 10 pg for E. canis and B. gibsoni.
• The strength of agreement for diagnostic sensitivity and specificity of multiplex PCR assay (n = 61) for detection of A. platys, B. gibsoni, E. canis, H. canis and B. vogeli revealed to be ‘very good’ taking singleplex PCR as standard test.
• The mPCR survey recorded 31.56% prevalence of tick-borne haemoparasitic infections in dogs with history/presence of tick infestation in 8 selected districts of Odisha (n = 320).

## Graphical abstract

**Figure:**
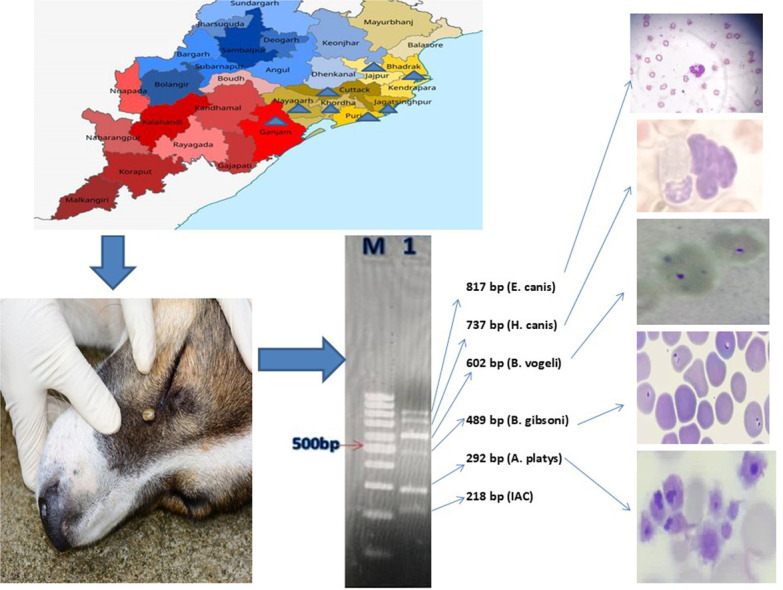

## 1 Introduction

India is home to an estimated 62 million street dogs and 19.5 million owned dogs (Brill et al., 2024). However, these population are not restricted to specific geographical boundaries owing to the movements of pets from one region to another, deployment of working dogs in defense organizations and dog trading at the national and international markets, that facilitates relocation of the tick vectors. The tropical climate, high humidity and abundant rainfall, as experienced in coastal states of eastern India, provide an optimal environment for the survival and proliferation of the tick vectors (Thomas et al., 2022). *Rhipicephalus sanguineus* stands out as the most prevalent vector among the diverse ticks infesting dogs in India, followed by *Haemaphysalis* species (Abd Rani et al., 2011). These vector ticks sustain and propagate many serious canine haemoparasitic diseases of protozoan origin, namely babesiosis and hepatozoonosis, and rickettsial origin diseases such as ehrlichiosis and anaplasmosis. Multiple hemoparasites, specifically *Babesia vogeli, Ehrlichia canis, Anaplasma platys, and Hepatozoon canis,* share the same brown dog tick, *R. sanguineus s. l.,* as a primary vector*.* Conversely, the transmission of *Babesia gibsoni* is attributed to both *Haemaphysalis* and *Rhipicephalus* ticks (Bilwal et al., 2017; Manoj et al., 2020). The alternative routes for pathogen spread other than tick-mediated transmission, also include vertical transmission, blood transfusions, and injuries sustained in fights (Hasani et al., 2024; Karasová et al., 2022; Latrofa et al., 2016).

In Indian subcontinent, canine babesiosis is accounted to two major species viz *Babesia vogeli* and *B. gibsoni* (Mittal et al., 2017). *B. vogeli* usually causes subclinical infections with a low parasitemia in adult dogs, but it takes a clinical course due to severe anemia in puppies and grey hounds (Singla et al., 2016). On the other hand, *B. gibsoni* is highly pathogenic and causes high grade fever, pale mucous membranes, jaundice, splenomegaly, weakness, thrombocytopenia, pigmenturia and collapse, associated with intra- and extra-vascular haemolysis, hypoxic injuries and systemic inflammation (Irwin, 2009). Canine hepatozoonosis is a systemic infection, caused by *Hepatozoon canis* exhibiting a wide array of signs such as fever, inappetence, weight loss, anaemia, hyperglobulinemia, often resulting in hepatitis, pneumonia and glomerulonephritis compounded by its unique transmission route, which involves ingestion rather than tick bites (Baneth et al., 2003). *Ehrlichia canis* is responsible for canine monocytic ehrlichiosis, characterized by a clinical spectrum ranging from mild, nonspecific symptoms to severe, sometimes fatal pancytopenia and hemorrhagic (Mylonakis & Theodorou, 2017). *Anaplasma platys* induces canine infectious cyclic thrombocytopenia, which may undermine the host’s immune system in subclinical form, and predispose to co-infections and more severe clinical outcomes (Harrus et al., 2005). The frequent co-infection of dogs with multiple Tick-borne pathogens (TBPs) is facilitated by their overlapping tick vectors, making diagnosis and management more complex. Such coinfections often aggravate clinical manifestations, complicate differential diagnosis, diminish treatment efficacy, and contribute to poor prognoses (Kledmanee et al., 2009). Diagnostic challenges are compounded by limitations of traditional methods. The peripheral blood smear examination via microscopy, although considered as a gold standard, often fails to detect low-parasitemia or chronic carrier states (Kordic et al., 1999). Similarly, serological techniques suffer from cross-reactivity, inability to distinguish between past and present infections, and increased cost, thus limiting their practical utility (Waner et al., 1998). Nucleic acid-based detection of parasites in clinical samples with higher sensitivity and specificity, such as polymerase chain reaction (PCR), sequencing and phylogenetic analysis have been employed globally for the diagnosis of active and ongoing infections and speciation of the isolates, respectively (Pirata et al., 2015).

Conventional multiplex PCR (mPCR) assays, capable of detecting multiple pathogens and co-infections in a single reaction, have been advocated as rapid, cost-effective, and scalable tools for epidemiological surveillance of canine tick-borne diseases (TBDs) (Liu et al., 2015), However, limitations such as lower sensitivity compared to conventional PCR and vulnerability to false-negative results due to amplification inhibitors highlight areas requiring methodological refinement (Azhahianambi et al., 2018; Kaur et al., 2020). The incorporation of internal amplification controls (IACs), a proven strategy to monitor integrity of the assay and mitigate the risk of false negatives, enhance the overall effectiveness of the amplification in each tube (Rosenstraus et al., 1998).

Multiplex PCR-based assays along with sensitivity comparison have been used earlier for the detection and epidemiological studies on haemoparasitic diseases in dogs (Kaur et al., 2020; Singh et al., 2024). However, reports are lacking till date, on conventional end-point multiplex PCR assay for the concurrent detection and differentiation of five major canine tick-borne hemoparasites, namely *Ehrlichia. canis, Babesia gibsoni, Babesia vogeli, Hepatozoon canis* and *Anaplasma platy*s with integrated internal control. Moreover, there is a critical lack of molecular epidemiological data that hinders developing effective disease control measures and impedes animal health surveillance efforts in Odisha, India. The present study was aimed to bridge these gaps by deploying a novel internally controlled hexaplex PCR assay for the simultaneous detection and characterization of vital tick-borne pathogens in dogs, and to provide valuable insights into the biological diversity and molecular epidemiology of these pathogens by sequence comparison with reference strains from diverse geographic localities.

## 2 Materials and method

### 2.1 Study area

**Fig. 1:**
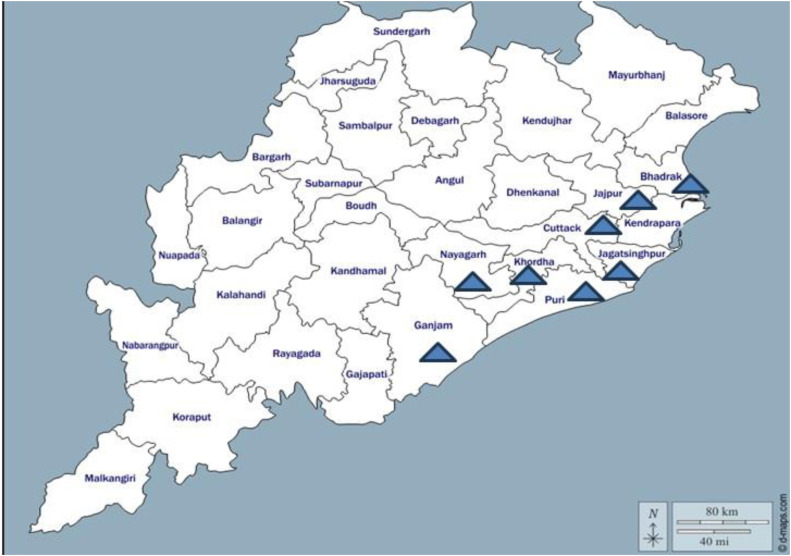
Map of Odisha with districts included in the investigation marked by blue triangle.

Odisha (17°49′–22°34′ N, 81°27′–87°29′ E), situated along India’s eastern seaboard, encompasses a geographical area of 155,707 km². The coastal belt of the state is classified under the Tropical Savanna (Aw) climate type according to the Köppen–Geiger system, typified by pronounced wet and dry seasons, high temperatures, and substantial monsoon rainfall (Kottek et al., 2006). For the present epidemiological investigation, eight coastal districts representing the coastal agro-climatic zone of Odisha, were selected namely Khordha,Cuttack, Puri, Ganjam, Nayagarh, Bhadrak, Jajpur, and Jagatsingpur (Fig. 1)**.**

### 2.2 Sampling of animal

The investigation was conducted from January to December 2024 on a cohort of 320 dogs drawn irrespective of sex, age, or breed, each exhibiting current or historical tick infestation, with or without clinical manifestations of tick-borne haemoparasitic diseases (TBD), such as fever, lethargy, limb weakness, respiratory distress, lymphadenomegaly, ocular abnormalities, and haemorrhagic disorders. Dogs were arbitrarily sampled from eight districts those were presented to the Veterinary Clinical Complex, College of Veterinary Science and Animal Husbandry, Odisha University of Agriculture and Technology, Bhubaneswar. Whole blood was aseptically collected from the cephalic or saphenous veins into EDTA vials by registered veterinarians, following informed consent from owners and in accordance with the blood collection guidelines of the Committee for Control and Supervision of Experiments on Animals (CCSEA). Thin blood smears were prepared immediately, and the remaining samples were stored at −80 °C until subsequent DNA extraction for molecular analyses.

### 2.3 Microscopy

A thin blood smear was prepared and subsequently stained with 10% Giemsa stain solution and viewed under oil immersion objective lens to detect the presence of large piroplasm of size around 5 µm * 2.5 µm of *B. vogeli and* small piroplasm of size around 2 × 1.5 µm of *B. gibsoni* in the erythrocytes, capsulated ellipsoidal gamonts with large central nucleus of *H. canis* in the neutrophils or monocytes, round or ovoid morulae of *E. canis* in monocytes or lymphocytes and round or ovoid shaped morulae of *A. platys* within thrombocytes for evidence infection (Soulsby, 1982). At least 100 oil immersion fields were examined before declaring the sample as negative.

### 2.4 Molecular analysis

Genomic DNA was extracted from whole blood samples collected from each dog using the DNeasy® Blood and Tissue Kit (Qiagen, Hilden, Germany), following the manufacturer’s protocol with minor modifications in the input blood volume (200 µl) and final elution volume (50 µl). The quality and purity of the eluates were assessed using a nano-spectrophotometer, and the DNA samples were stored at −80 °C until further analysis.

Conventional singleplex PCR assays were first optimized individually for each hemoparasite using species-specific primer pairs. Subsequently, a multiplex PCR assay was standardized to enable the simultaneous detection of *Babesia vogeli, Babesia gibsoni, Hepatozoon canis, Ehrlichia canis* and *Anaplasma platys*, as well as their possible co-infections, within a single-tube reaction. Highly parasitemic, microscopically positive samples, confirmed by PCR assay, were used as positive controls, whereas DNA extracted from an infection-free neonatal puppy served as the negative control, and a negative control was included in every PCR run to monitor contamination. Details of the oligonucleotide primers employed in this study are provided in Table 1.

**Table 1:** The primer sequences used in singleplex and multiplex PCR assay.

Sl no	Parasite	Sequence	Size	Target gene	Reference
1	*E. canis*	ECF5ʹ CAGCCACACTGGAACTGAGA 3ʹ ECR5ʹ GAGTGCCCAGCATTACCTGT 3ʹ	817bp	16S ribosomal RNA gene	(Chen et al., 2012)
2	*H. canis*	HEPF5ʹCCTGGCTATACATGAGCAAAATCTCAACTT 3ʹ HEPR 5ʹCCAACTGTCCCTATCAATCATTAAAGC 3ʹ	737bp	18S ribosomal RNA gene	(Kledmanee et al., 2009)
3	*B. vogeli*	BCV F 5ʹGTGAACCTTATCACTTAAAGG 3ʹ BCV R5ʹCAACTCCTCCACGCAATCG 3ʹ	602bp	18S ribosomal RNA gene	(Kaur et al., 2020)
4	*B. gibsoni*	BAGF5ʹ TGGGGTTTTCCCCTTTTTAC 3ʹ BAGR5ʹ TTC AGC CTT GCG ACC ATA C 3ʹ	489bp	18S ribosomal RNA gene	(Sathish et al., 2021)
5	*A. platys*	APF5’ GCCTTATGGGTCACCAGAGA 3′ APR5’ TTCAACTTCACGCCTCATTG 3′	292bp	Heat shock protein GroELgene	(Sathish et al., 2021)
6	Internal amplification control	β-actin F5ʹ CTGTCCCTGTATGCCTCT 3ʹ β-actin R5ʹATGTCACGCACGATTTCC 3ʹ	218 bp	canine β-actin gene	(Chen et al., 2014)

For singleplex PCR, a 20 μl reaction volume was standardized, comprising 0.5 μl of each primer (10 μM), 10 μl of 2X Multiplex Master Mix (Qiagen, Hilden, Germany), 8 μl of nuclease-free water, and 1 μl of template DNA. The thermal cycling conditions consisted of an initial activation at 95 °C for 15 min, followed by 35 cycles of denaturation at 94 °C for 30 s, annealing at 59–62 °C for 90 s, and extension at 72 °C for 90 s, with a final extension step at 72 °C for 10 min.

For multiplex PCR, the reaction mixture (50 μl total volume) contained 1 μl of each parasite-specific primer (five pairs, each at 10 μM) in addition to a primer pair targeting the canine β-actin gene as an internal control. The mixture further included 25 μl of Multiplex Master Mix (2X) (Qiagen, Hilden, Germany),5 μl of template DNA, and nuclease-free water to reach the final volume. Primer concentrations and annealing temperature were empirically optimized to achieve robust and specific amplification of all targets. The cycling profile comprised an initial activation at 95 °C for 15 min, followed by 35 cycles of denaturation at 94 °C for 30 s, annealing at 60 °C for 90 s, and extension at 72 °C for 90 s, with a final extension at 72 °C for 10 min.

All amplifications were performed in a Thermal Cycler (T100TM, Bio-Rad, Hercules, CA, USA). PCR amplicons were resolved by horizontal electrophoresis on 1.5% agarose gels prepared in Tris–acetate–EDTA (TAE) buffer and containing ethidium bromide (Himedia, India). Gels were run at 85 V for 60 min alongside a 100 bp DNA ladder to estimate product sizes. Amplification products were visualized and documented under UV light gel documentation system (Gel DocTM EZ Imager, Bio-Rad, Hercules, CA, USA).

Singleplex PCR products that yielded intense, distinct bands for each hemoparasite were selected for downstream sequencing. Target bands were excised and purified using the QIAquick® Gel Extraction Kit (Qiagen, Hilden, Germany), following the manufacturer’s guidelines, and purified products were outsourced for bidirectional Sanger sequencing (Eurofins Laboratories, Bangalore). The resulting nucleotide sequences were aligned and edited using MEGA 12 software, and representative sequences were deposited in the GenBank database, from which accession numbers were obtained: PV330100 (*A. platys*), PV329704 (*B. gibsoni*), PV329651 (*B. vogeli*), PV324962 (*E. canis*) and PV613156 (*H. canis*).

Multiple sequence alignment was carried out using the MUSCLE algorithm, and the sequences generated in this study were compared with homologous sequences retrieved from GenBank using the nucleotide BLAST (nBLAST) tool available at the National Center for Biotechnology Information (NCBI). Phylogenetic analyses were performed on the aligned nucleotide datasets using the maximum likelihood method with 1000 bootstrap replicates implemented in MEGA 12 (Kumar et al., 2024).

### 2.5 Analytical sensitivity of PCR assays

The parasite specific DNA fragments were amplified from the positive control genome samples, gel purified using QIAquick® Gel Extraction Kit (Qiagen, Hilden, Germany), and the purified DNA concentration was measured in the eluted DNA by a nano-spectrophotometer. The stock solution of DNA was serially diluted ten-fold (1ng/μl to 1 fg/μl) with nuclease free water and used as template in 20μl singleplex PCR test to estimate the threshold sensitivity level of the assay (Bilgiç et al., 2013). The amplified PCR products were resolved in 1.5% agarose gel containing ethidium bromide and subsequently, visualized and documented. In addition, equal quantities of DNA representing these five pathogens, were mixed and 10-fold serially diluted to evaluate the sensitivity of multiplex PCR assay. To avoid detection constraint, 1 ng/μl DNA solution from the negative control dog was used for serial dilutions of this DNA mixture, which also acted as working template for IAC (canine β-actin) primers (Ganguly et al., 2020).

### 2.6 Assessment of diagnostic sensitivity and specificity from controls and field samples

Sensitivity of an assay in comparision to a standard test = (True Positives)/(True Positives+False Negatives)

Specificity of an assay in comparision to a standard test = (True Negatives)/(True Negatives +False Positives) (Naeger et al., 2013)

The genomic DNA samples isolated from the positive control dogs for each parasite (n = 2) were used to validate the diagnostic efficiency of the multiplex PCR assay assuming the singleplex PCR as the standard test. Further, 61 random samples from field, with history of tick infestation, were used for both singleplex and multiplex PCR assays, targeting the specific parasite,to evaluate the sensitivity and specificity of the developed multiplex assay. Later, selected mPCR amplified products from field samples (2 each for a parasite species) were also sequenced for further confirmation.

### 2.7 Statistical analysis

The statistical analyses were performed using SPSS software, version 22 (IBM Corp., Armonk, NewYork, USA). The newly developed multiplex PCR assay was validated by screening 61 canine blood DNA samples collected from field cases, and its performance was compared with that of the corresponding singleplex PCR assays through estimation of McNemar’s test *p*-value, Cohen’s kappa coefficient, specificity, and sensitivity, together with their 95% confidence intervals (CI).The associations between putative risk factors and the prevalence of individual tick-borne haemoparasitic infections were assessed using univariate analysis and multivariate logistic regression analysis, and probability of error up to 5% was considered acceptable (P < 0.05). The selection of variables to be included in the multivariate model, was carried through univariate analysis to assess the association between each variable and variables for which p > 0.3 in the univariate analysis, were excluded from further analysis.The risk factors analyzed included locality (Khordha, Cuttack, Puri, Ganjam, Nayagarh, Bhadrak, Jajpur, and Jagatsinghpur), sex (male or female), age group (< 1 year, 1–5 years, or > 5 years), breed (non-descript, Golden Retriever, Labrador Retriever, Spitz, or others), tick infestation status, acaricide treatment (none, within the preceding 2 months, or >2 months prior), and level of outdoor activity (none, limited, or active)

## 3 Result

### 3.1 Microscopical examination

The microscopic examination of blood smears from 320 dogs across eight coastal districts in Odisha revealed an overall prevalence of 12% for tick-borne infections. The he highest prevalence was recorded for *Babesia gibsoni* infection (8.1%) and the lowest for *Babesia vogeli* (0.3%). Notably, there were no microscopically confirmed instances of concurrent or mixed infections observed within this cohort.

### 3.2 Analytical sensitivity of singleplex and multiplex PCR assay

Singleplex PCR assays generated distinct amplicons sized 817 bp (*E. canis*), 737 bp (*H. canis*), 602 bp (*B. vogeli*), 489 bp (*B. gibsoni*), and 292 bp (*A. platys*). The newly developed hexaplex PCR assay efficiently amplified all these fragments concurrently, along with an internal amplification control (IAC) yielding a 218 bp product, without generating any non-specific products (Supplementary Fig. 1 A-F). Detection threshold assessment established that singleplex PCR detected *A. platys, B. gibsoni, E. canis,* and *B. vogeli* DNA at concentrations as low as 0.01 pg. Conversely, the multiplex assay showed a detection threshold of 0.1 pg for *A. platys* and *B. vogeli*, 10 pg for *E. canis* and *B. gibsoni*. However, for *H. canis,* both the PCR assays revealed a value of 0.1 pg (Table 2).

**Table 2:** Detection limit of singleplex and multiplex PCR assay.

Parasite	Singleplex PCR		Multiplex PCR	
	Dilution rate	DNA conc. (pg /μl)	Dilution rate	DNA conc. (pg /μl)
*A. platys*	10^–5^	0.01	10^–4^	0.1
*B. gibsoni*	10^–5^	0.01	10^–2^	10
*E. canis*	10^–5^	0.01	10^–2^	10
*H. canis*	10^–4^	0.1	10^–4^	0.1
*B. vogeli*	10^–5^	0.01	10^–4^	0.1

### 3.3 Assessment of diagnostic sensitivity and specificity from controls and field samples

The validation of the standardized multiplex PCR assay on positive control samples (2 each) revealed the diagnostic specificity and sensitivity as 100% (Supplementary Fig. 2 A-E). Further, validation on 61 field samples revealed the diagnostic sensitivity (95% CI) of multiplex PCR assay in the detection of *A. platys, B. gibsoni, E. canis, H. canis* and *B. vogeli* as 90.5% (87.7% to 93.2%), 94.12% (91.4%to 96.8%), 87.5% (83.4% to 91.5%), 83.33% (71.2% to 95.4%), 100% (98.04% to 100%), respectively. The diagnostic specificity (95% CI) of multiplex PCR assay in the detection of *A. platys* and *H. canis* was evaluated as 97.5% (96.7% to 98.3%) and 98.18% (96.96% to 99.4%), respectively, whereas that of B. *gibsoni, E. canis and B. vogeli* was detected as 100% (98.04% to 100%). Statistical analysis using McNemar's test indicated that there was no significant difference between singleplex and multiplex PCR assays in detection of *A. platys, B. gibsoni, H. canis, B. vogeli* (*p* = 1.000) and *E. canis* (*p* = 0.5)*.* Kappa values indicated very good agreement between the two PCR modalities. (Table 3).

**Table 3:** Comparative analysis of singleplex and multiplex PCR assay.

*Anaplasma platys*	Singleplex PCR		McNemar's Test (Two tailed) p value	Kappa value ±S.E. (agreement)	Sensitivity	95% CI	Specificity	95% CI	
Multiplex PCR	Negative	Positive	Total						
Negative	39	2	41	1	0.89±0.062 (very good)	90.50%	87.7 to 93.2%	97.50%	96.7% to 98.3%
positive	1	19	20						
Total	40	21	61						
*Babesiagibsoni*	singleplex PCR								
Multiplex PCR	Negative	Positive	Total	1	0.958±0.041 (very good)	94.12%	91.4 to96.8%	100	98.04 to 100%
Negative	44	1	45						
positive	0	16	16						
Total	44	17	61						
*Ehrlichia canis*	singleplex PCR								
Multiplex PCR	Negative	Positive	Total	0.5	0.912±0.061 (very good)	87.50%	83.4 to 91.5%	100	98.04 to 100%
Negative	45	2	47						
positive	0	14	14						
Total	45	16	61						
*Hepatozoon canis*	singleplex PCR								
Multiplex PCR	Negative	Positive	Total	1	0.815±0.127 (very good)	83.33%	71.2 to 95.4%	98.18%	96.96 to 99.4%
Negative	54	1	55						
positive	1	5	6						
Total	55	6	61						
*Babesia vogli*	singleplex PCR								
Multiplex PCR	Negative	Positive	Total	1	1 ± 0 (very good)	100	98.04 to 100%	100	98.04 to 100%
Negative	59	0	59						
positive	0	2	2						
Total	59	2	61						

CI: confidence interval.

### 3.4 Field evaluation of multiplex PCR

Employing the multiplex PCR assay on field samples revealed a substantially higher prevalence of tick-borne haemoparasitic infections at 31.56%, indicative of enhanced detection sensitivity compared to microscopy. Single-pathogen infections were detected in 20.93% of dogs, whereas multiple-pathogen coinfections accounted for 10.62% . Amongst single infections, *B. gibsoni* remained the most prevalent (10.3%), whereas lowest was recorded in case of *B. vogeli* (0.6%). Among coinfected dogs, dual infections comprised 9.4%, and triple infections were observed in 1.2%. Overall, *A. platys* displayed the highest overall prevalence at 16.3%, while *B. vogeli* remained the least prevalent at 0.9%. The multiplex PCR diagnostic sensitivity was significantly higher than microscopy for all hemoparasites except *B. vogeli* (Table 4).

**Table 4:** Microscopy and multiplex PCR assay of field samples.

Parasite	Microscopy			mPCR		
	SI	MI	Total	SI	MI	Total
*A. platys*	6 (1.9%)	0	6 (1.9%)	23 (7.2%)	29 (9.1%)	52 (16.3%) *
*B. gibsoni*	26 (8.1%)	0	26 (8.1%)	33 (10.3%)	13 (4.1%)	46 (14.4%) *
*B. vogeli*	1 (0.3%)	0	1 (0.3%)	2 (0.6%)	1 (0.3%)	3(0.9%)
*E. canis*	5 (1.6%)	0	5 (1.6%)	6 (1.9%)	23 (7.2%)	29 (9.1%) *
*H. canis*	3 (0.9%)	0	3 (0.9%)	3 (0.9%)	6 (1.9%)	9 (2.8%) *
BG+EC	0	0	0	0	5 (1.6%)	5 (1.6%)
AP+EC	0	0	0	0	14 (4.4%)	14 (4.4%)
AP+HC	0	0	0	0	5 (1.6%)	5 (1.6%)
AP+BG	0	0	0	0	5 (1.6%)	5 (1.6%)
AP+BV	0	0	0	0	1 (0.3%)	1 (0.3%)
AP+BG+EC	0	0	0	0	3 (0.9%)	3 (0.9%)
AP+HC+EC	0	0	0	0	1 (0.3%)	1 (0.3%)

SI: Single infection, MI: Mixed infection.

BG+EC - *Babesia gibsoni* with *Ehrlichia canis*; AP+EC - *Anaplasma platys* with *Ehrlichia canis;* AP+HC - *Anaplasma platys* with *Hepatozoon canis;* AP+BG - *Anaplasma platys* with *Babesia gibsoni;* AP+ BV - *Anaplasma platys* with *Babesia vogli*; AP + BG + EC – *Anaplasma platys* with *Babesia gibsoni* and *Ehrlichia canis;* AP+HC+EC- *Anaplasma platys* with *Hepatozoon canis* and *Ehrlichia canis*.

The figures in percent in parenthesis indicates prevalence (n = 320).

⁎ *p* < 0.05; McNemar's Test (Two tailed) p value.

### 3.5 Multiplex PCR analysis, risk factors and prevalence

The association of risk factors with prevalence of various tick-borne pathogens in tick exposed dogs was depicted in Table 5, Table 6. The analysis of risk factors by both univariate and multivariate analysis indicated non-significant prevalence variation amongst districts except in Khordha, where infection with *B. gibsoni* was significantly elevated. Seasonal variation significantly influenced the prevalence of *B. gibsoni,* and *H. canis*, with summer months exhibiting the highest infection rates. On the contrary, seasonal multivariate analysis depicted the higher odds of infection of *A. platys* associated with rainy season while the risk association with infection in summer season being non-significant. This pattern did not apply to *E. canis* and *B. vogeli*, whose prevalence was unaffected by season.

**Table 5:** Distribution of canine tick borne haemoparasitic infections with respect to various risk factors.

Risk factor	Parameter	n	Prevalence				
			*A. platys*	*B. gibsoni*	*B. vogeli*	*E.canis*	*H. canis*
Place	Khordha	107	21(19.6%)	26(24.3%)	2(1.9%)	11(10.3%)	3(2.8%)
	Cuttack	43	11(25.6%)	6(14%)	0	4(9.3%)	4(9.3%)
	Puri	32	6(18.8%)	3(9.4%)	0	2(6.3%)	1(3.1%)
	Ganjam	32	4(12.5%)	3(9.4%)	0	6(18.8%)	0
	Nayagad	27	2(7.4%)	2(7.4%)	0	0	0
	Bhadrak	26	2(7.7%)	3(11.5%)	1(3.8%)	1(3.8%)	0
	Jajpur	27	2(7.4%)	1(3.7%)	0	2(7.4%)	1(3.7%)
	Jagatsingpur	26	4(15.4%)	2(7.7%)	0	3(11.5%)	0
Irrespective of sampling districts	320	52(16.3%)	46(14.4%)	3(0.9%)	29(9.1%)	9(2.8%)	
Pearson χ^2^ value(Pvalue)		8.641(0.279)	14.544(0.042) *	5.138(0.643)	7.979(0.334)	9.12(0.193)	
Season	Summer	105(32.81%)	25(23.8%)	25(23.8%)	1(1%)	13(12.4%)	7(6.7%)
	Rainy	148(64.35%)	23(15.5%)	11(7.4%)	0	10(6.8%)	1(0.7%)
	Winter	67(20.93%)	4(6.0%)	10(14.9%)	2(3%)	6(9%)	1(1.5%)
Pearson χ^2^ value(P value)		9.666(0.008)*	13.005(0.001)*	3.959(0.138)	2.359(0.307)	8.606(0.014)*	
Breed	Nondescript	128(40%)	19(14.8%)	20(15.6%)	1(0.8%)	13(10.2%)	2(1.6%)
	Golden retriever	34(10.63%)	6(17.6%)	6(17.6%)	0	2(5.9%)	2(5.9%)
	Labrador	67 (20%)	9(13.4%)	5(7.5%)	1(1.5%)	6(9%)	3(4.5%)
	Spitz	59(18.43%)	13(22%)	8 (13.6%)	0	6(10.2%)	1(1.7%)
	Other	32(10%)	5(15.6%)	7 (21.9%)	1(3.1%)	2(6.3%)	1(3.1%)
Pearson χ^2^ value(Pvalue)		2.085(0.72)	4.553(0.336)	2.785(0.594)	0.99(0.91)	2.865(0.581)	
Sex	Female	160(50%)	29(18.1%)	26(16.3%)	1(0.6%)	18(11.3%)	4(2.5%)
	Male	160(50%)	23(14.4%)	20(12.5%)	2(1.3%)	11(6.9%)	5(3.1%)
Pearson χ2 value(P value)		0.827(0.363)	0.914(0.339)	0.336(0.562)	0.858(0.373)	0.114(0.735)	
Age	<1 year	61(19.06%)	10(16.4%)	11(18%)	2(3.3%)	3(4.9%)	2(3.3%)
	1–5 year	195(60.94%)	32(16.4%)	25(12.8%)	0(%)	20(10.3%)	7(3.6%)
	>5 year	64(20%)	10(15.6%)	10(15.6%)	1(1.6%)	6(9.4%)	0
Pearson χ^2^ value(P value)		0.023(0.989)	1.127(0.569)	5.715(0.05) *	1.616(0.446)	2.332(0.312)	
Tick infestation	Yes	73(22.81%)	32(43.8%)	23(31.5%)	1(1.4%)	13(17.8%)	7(9.6%)
	No	247(77.19%)	20(8.1%)	23 (9.3%)	2 (0.8%)	16 (6.5%)	2(0.8%)
Pearson χ^2^ value(P value)		52.88(≤0.001) *	22.55(≤0.001) *	0.19 (0.663)	8.778(0.003) *	15.889(≤0.001) *	
Anti-tick treatment	Nil	50(15.62%)	15(30.8%)	16(32%)	1(2%)	8(16%)	1(2%)
	Beyond 2 months	238(74.37%)	37(15.5%)	29(12.18%)	2(0.8%)	20(8.4%)	8(3.4%)
	Within 2 months	32(10%)	0	1(3.1%)	0	1(3.1%)	0
Pearson χ^2^ value (P value)		13.242(0.001) *	18.597(≤0.001) *	0.935(0.627)	6.126(0.047) *	1.309(0.52)	
Outside activity	Nil	130(56.52%)	2(1.5%)	7(5.4%)	2(1.5%)	4(3.1%)	1(0.8%)
	Active	100(31.25%)	12(12%)	16(16%)	0	8(8%)	1(1%)
	Limited	90(28.12%)	38(42.2%)	23(25.6%)	1(1.1%)	17(18.9%)	7(7.8%)
Pearson χ^2^ value(P value)		66.61(≤0.001)*	17.89(≤0.001) *	1.481(0.477)	16.33(≤0.001)*	13.775(0.001)*	

The figures in percent in parenthesis indicates prevalence (n = 320), n= Number of dogs.

⁎ Significant at *p* ≤ 0.05.

**Table 6:** Multivariate logistic regression analysis of risk factors associated with tick-borne pathogen infection in dogs.

Risk factor	Parameter	OR (P value)				
		*A. platys*	*B. gibsoni*	*B. vogeli*	*E.canis*	*H. canis*
Place	Khordha	1.04(0.29)	2.43(0.04)*	-	-	2.49(0.41)
	Cuttack	0.48(0.33)	0.27(0.06)	-	-	0.54(0.71)
	Puri	0.61(0.56)	0.39(0.2)	-	-	0.25(0.34)
	Ganjam	0.81(0.83)	0.3(0.18)	-	-	0.95(0.61)
	Nayagad	0.47(0.5)	0.34(0.17)	-	-	0.29(0.28)
	Bhadrak	0.56(0.57)	0.24(0.21)	-	-	0.46(0.67)
	Jajpur	0.95(0.96)	0.37(0.65)	-	-	0.93(0.94)
Season	Summer	0.44(0.22)	4.76(0.03)*	1.71(0.09)	1.02(0.98)	1.08(0.04)*
	Rainy	4.51(0.04)*	0.59(0.29)	0.01(0.99)	0.22(0.48)	0.04(0.08)
Age	<1 year	-	-	1.21(0.05)*	-	-
	1–5 year	-	-	0	-	-
Tick infestation	Yes	4.27(0.002)*	3.43(0.02)*	-	2.18(0.05)*	14.92(0.02)*
Anti-tick treatment	Nil	24.72(0.001)*	18.93(0.001)*	-	8.34(0.01)*	-
	Beyond 2 months	4.49(0.072)	2.81(0.04)*	-	1.21(0.09)	-
Outside activity	Active	42.7(0.001)*	1.3(0.07)	-	4.2(0.01)*	1.02(0.34)
	Limited	19.06(0.001)*	19.7(0.002)*	-	1.78(0.5)	8.83(0.01)*

OR: Odds ratio.

⁎ Significant at *p* ≤ 0.05.

Host factors such as breed, sex, and age generally showed no significant association with infection,except a significant susceptibility of <1 year dog to *B. vogeli* infection. Out of 320 dogs, 73 dogs were found to have ticks upon them during sampling. Tick infestation was a highly significant risk factor correlating with increased infection rates by *A. platys, B. gibsoni, E. canis,* and *H. canis* (p < 0.01) compared to history of exposure. Anti-tick treatment also significantly reduced disease incidence except for *B. vogeli*, with dogs untreated with acaricides exhibiting the highest infection rates except *H. canis*. Dogs treated with acaricide within the past two months displayed negligible *A. platys, B. vogeli,* and *H. canis* infections and the lowest incidences of *B. gibsoni* and *E. canis*.

Additionally, outdoor activity significantly impacted infection risks. Dogs with no outdoor exposure had the lowest prevalence for haemoparasitic infections, whereas those with limited outdoor activity had the highest, except for *H. canis in which feral dogs exhibited heightened infection. B. vogeli infection was not significantly affected with outside activity.*

### 3.6 Analysis of clinical status of tick-borne pathogen positive dogs

Clinical status of dogs was accessed in the mPCR positive dogs for different TBP infections and co-infections on the basis of presence of clinical symptoms such as inappetance, pyrexia, lymphnode enlargement, lethargy, pallor of mucosa, congested mucosa, dermal petechiae, jaundice, emaciation, vomition, diarrohea, melena, pigmenturia, epistaxis, ocular abnormality, ascitis, cough, hindleg paresis and seizure. In cases of single infections, the highest prevalence of asymptomatic dogs was observed in those infected with *H. canis* (66.7%), while *B. gibsoni* infection revealed the highest proportion of clinically affected dogs (64%). Co-infections exacerbated disease severity while triple infections resulted in 100% clinical affection (Table 7).

**Table 7:** Clinical status of tick-borne pathogen positive dogs.

Parasite	Total no of positives detected by mPCR	Clinical sign	
		Present	Absent
*A. platys*	29	12(41.37%)	17(58.62%)
*B. gibsoni*	25	16(64%)*	9(36%)
*B. vogeli*	2	1(50%)	1(50%)
*E. canis*	6	3(50%)	3(50%)
*H. canis*	3	1(33.3%)	2(66.7%)
BG+EC	5	4(80%)*	1(20%)
AP+EC	14	11(78.57%)*	3(21.42%)
AP+HC	5	3(60%)	2(40%)
AP+BG	5	4(80%)*	1(20%)
AP+BV	1	1(100%)	-
AP+BG+EC	3	3(100%)	-
AP+HC+EC	1	1(100%)	-

BG+EC - *Babesia gibsoni* with *Ehrlichia canis;* AP+EC - *Anaplasma platys* with *Ehrlichia canis*; AP+HC - *Anaplasma platys* with *Hepatozoon canis*; AP+BG - *Anaplasma platys* with *Babesia gibsoni*; AP+ BV - *Anaplasma platys* with *Babesia vogli*; AP + BG + EC – *Anaplasma platys* with *Babesia gibsoni* and *Ehrlichia canis*; AP+HC+EC- *Anaplasma platys* with *Hepatozoon canis* and *Ehrlichia canis*.

⁎ Significant at p ≤ 0.05 revealed by Chi-square analysis.

### 3.7 Phylogenetic analysis

The phylogenetic analysis was performed based on partial sequences of 18S rRNA gene of *H. canis, B. gibsoni*, and *B. vogeli*; 16S rRNA gene of *E. canis* and HSP GroEL gene of *A. platys,* retrieved from the NCBI database. The sequence study of the Odisha isolates also revealed 99–100% similarity between various global isolates of these pathogens from dogs.

**Fig. 2:**
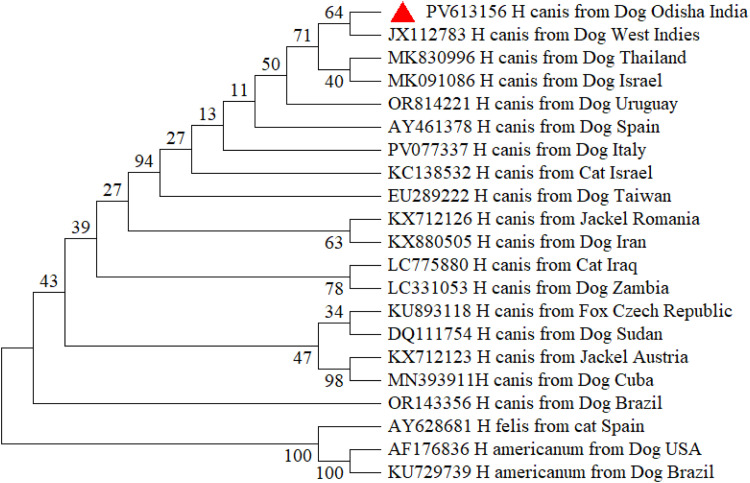
Phylogenetic analysis of *H. canis* based on partial sequence of 18 s rRNA gene. Evolutionary analysis was conducted on 1000 bootstrap replications using Maximum Likelihood method. Sequences are presented by GenBank accession number, pathogen species, host and country of origin.

**Fig. 3:**
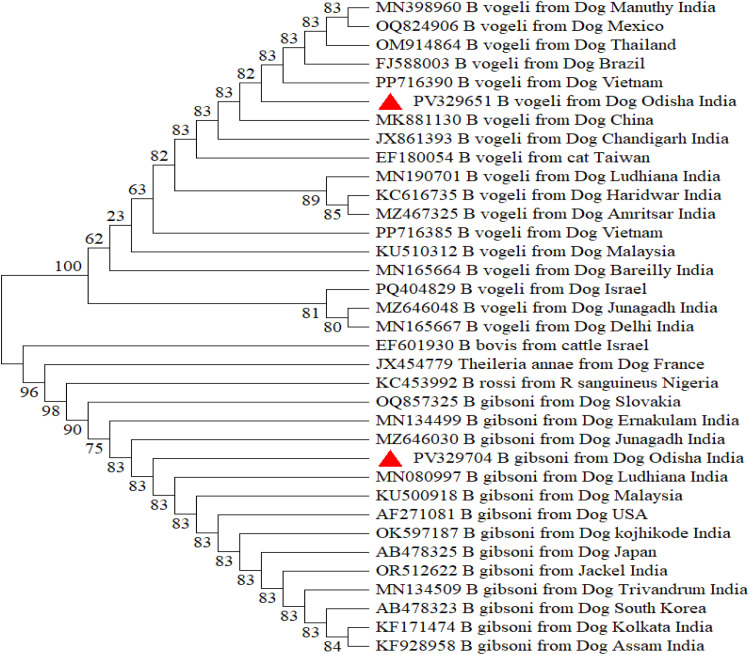
Phylogenetic analysis of *Babesia* spp*.* based on partial sequence of 18 s rRNA gene. Evolutionary analysis was conducted on 1000 bootstrap replications using Maximum Likelihood method. Sequences are presented by GenBank accession number, pathogen species, host and country of origin.

**Fig. 4:**
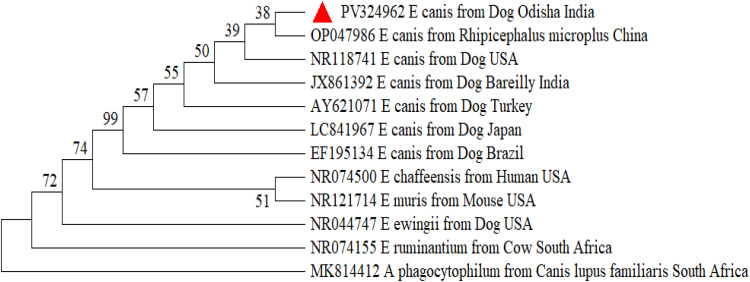
Phylogenetic analysis of *E. canis* based on partial sequence of 16 s rRNA gene. Evolutionary analysis was conducted on 1000 bootstrap replications using Maximum Likelihood method. Sequences are presented by GenBank accession number, host and country of origin.

**Fig. 5:**
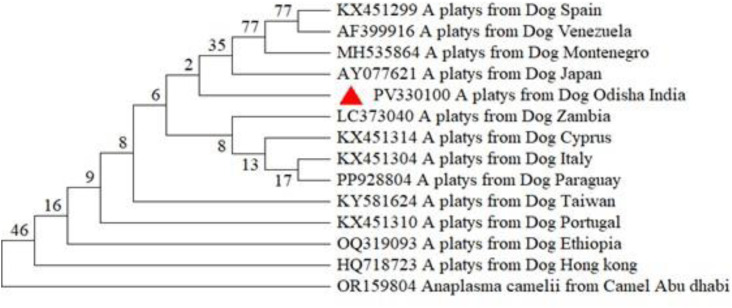
Phylogenetic analysis of *A. platys* based on partial sequence of GroEL gene*.* Evolutionary analysis was conducted on 1000 bootstrap replications using Maximum Likelihood method. Sequences are presented by GenBank accession number, host and country of origin.

The Odisha isolate of *Hepatozoon canis* formed a robust clade without any distinct sub-clustering, indicating close evolutionary affinity with isolates from various global regions, including nucleotide sequences of *H. canis*, isolated from dogs of West Indies (JX115783), Thailand (MK830996) and Israel (MK091086) with exclusion of the clade containing other species of *Hepatozoon i*ncluding *H. felis* and *H. americanum* (Fig. 2). The *Babesia* tree demonstrated two well-resolved clades separating *B. gibsoni* and *B. vogeli*, with Odisha isolates grouped firmly within respective species clades with bootstrap values >80%. Distinct interspecies separation from other *Babesia* spp. such as *B. rossi, B. bovis* and *Babesia vulpes (previously termed as Theileria annae*)confirmed accurate species identification of both *B. gibsoni* and *B. vogeli. B. gibsoni* isolates from Odisha exhibited high genetic similarity, mostly with the isolates from other parts of India; the same pattern was observed for *B. vogeli*. The Odisha isolate of *B. vogeli* was closely related with nucleotide sequences of *B. vogeli*, isolated from dogs of Vietnam (PP716390), China (MK881130) and Chandigarh (MN398960) and the isolate of *B. gibsoni* was closely related with Junagadh isolate (MZ646063) and Ludhiana isolate (MN080997) of India along with Malaysian isolate (KU500918) (Fig. 3). The Odisha isolate of *E. canis* formed a distinct clade with isolates from various countries and was found to be closely related with the USA isolate (NR118741) from dogs and the China isolate from *Rhipicephalus* tick (OP047986) and Bareilly isolate (JX861392). The clustering of *E. canis* sequences was distinct from other *Ehrlichia* species, including *E. chaffeensis, E. muris, E. ewingii*, and *E. ruminantium*, as well as *Anaplasma phagocytophilum*, confirming species-level resolution (Fig. 4). Similar pattern was observed for *A. platys* and was closely related to the nucleotide sequences of *A. platys*, isolated from dogs from Japan, Montenegro, and Zambia (GenBank: AY077621, MH535864, LC373040 respectively) and distant from *Candidatus Anaplasma camelii* isolate from Abu Dhabi (Fig. 5).

## 4 Discussion

This pioneering investigation delineates the successful development and application of a conventional end-point hexaplex PCR assay, facilitating simultaneous detection and differentiation of five principal tick-borne hemoparasites infecting dogs, namely *Anaplasma platys, Babesia gibsoni, Ehrlichia canis, Hepatozoon canis,* and *Babesia vogeli*, in Odisha, an eastern coastal state in India. To the best of current knowledge, this represents the inaugural molecular epidemiological assessment of these pathogens including analyses of their genetic diversity in this region that also encompassed the first documented report of *Anaplasma platys* in canines and first molecular evidence of *Hepatozoon canis* from Odisha. Although internally controlled conventional hexaplex PCR assays have been reported earlier for differentiation of human *Plasmodium* species (Chew et al., 2012), their application to canine tick-borne diseases (TBDs) remained unprecedented. Notably, two novel quadruplex TaqMan-based real-time PCR assays (qPCRs) were recently developed for detecting these five tick-borne pathogens (TBPs) alongside haemotropic *Mycoplasma* spp (Huggins et al., 2021). Although the TaqMan approach affords superior sensitivity along with quantification of parasites and mitigates false positives through double-labelled probes and the exonuclease activity of Taq polymerase, mutations within probe-binding sites may impede probe annealing and detection (Papin et al., 2004). Furthermore, conventional multiplex PCR (mPCR) assays, though less sensitive, still offer greater economic viability for large-scale screening in resource-limited settings prevalent in developing countries. In Indian scenario, epidemiological surveys employing mPCR assays in canines were confined to Tamil Nadu, Kerala, and Punjab (Azhahianambi et al., 2018; Jain et al., 2018; Kaur et al., 2020; Singh et al., 2024).Though conventional end point multiplex PCR assay offers the advantage of simultaneous detection of more than one parasitic DNA in a single tube, but the optimization of the assay remains challenging. Key issues include cross-hybridization among multiple primer pairs, amplicon size variations, and contamination risks during agarose gel electrophoresis, which tends to yield false positives. Moreover, low GC content of the primers and presence of both high and low aboundance targets (causing preferential amplification of certain specific targets) results in lower detection sensitivity of the assay (Elnifro et al., 2000). In this study, the assay was optimized through the selection of primers without any significant homology either internally or to one another, by utilising primers with nearly identical optimum annealing temperatures, limiting the GC content within 40–60% and elimination of cross-contamination during gel electrophoresis. Thereby, the assay was able to achieve maximum sensitivity and specificity, comparable to its singleplex assay counterparts without any significant difference. Despite the use of six primer sets in one reaction, including one set for IAC, the multiplex PCR was able to detect five hemoparasites at a higher resolution. It produced amplicons of comparable sizes without any non-specific amplification, allowing clear differentiation via agarose gel electrophoresis. Moreover, the co-amplification of the internal control alongside the target gene affirmed overall integrity of the reaction and further validated the result by discounting false negatives from PCR inhibitors. Further, the sequencing of PCR products also confirmed the specific amplification. Comparative analysis indicated a slight reduction in multiplex PCR sensitivity relative to singleplex assays, particularly a 10- to 100-fold increase in detection threshold for *A. platys, B. vogeli, B. gibsoni,* and *E. canis* whereas, *H. canis* sensitivity remained comparable. This trend is congruent with previous multiplex PCR diagnostic studies (Azhahianambi et al., 2018; Kaur et al., 2020) and competition between multiple primers for a finite quantity of reagents might be the contributing factor (Kundave et al., 2018).

The multiplex PCR assay demonstrated substantially superior diagnostic sensitivity relative to microscopy, identifying 31.56% infection prevalence compared to 12% by smear examination. This underscores the crucial advantage of molecular assays in detecting subclinical, mixed, and latent infections, which are frequently missed microscopically due to lower parasitemia and the transient nature of pathogen inclusions in blood cells (Dumler & Brouqui, 2004; Torina et al., 2012). Intriguingly, while microscopy suggested *B. gibsoni* as the dominant hemoparasite, molecular diagnostics revealed *A. platys* as most prevalent, consistent with its subclinical nature and the difficulty visualizing platelet inclusion bodies in early or low-grade infections (Ferreira et al., 2007).

The molecular epidemiological insights of this study differ from prior Indian TBD surveys reporting higher prevalence of *A. platys* in the circulation in contrast to the other places of India, such as *E. canis* (39.5%) in Delhi and *H. canis* in Mumbai (43.8%), Ladakh (24%) (Abd Rani et al., 2011); Tamil Nadu (37.8%) (Manoj et al., 2020); Punjab (Thomas et al., 2022); *B. gibsoni* in Mizoram and Tripura (43%) (Sarma et al., 2019). Such geographic variation likely reflects the differences in host population structures, sample sizes, climatic influences, and targeted genetic markers utilized in diagnostics (Do et al., 2021).

Coinfection prevalence was notable, with mixed infections in 10.62% of dogs, corroborating international reports that multiple TBD pathogen coexistence is common and can elicit complex clinical manifestations affecting diagnosis and treatment strategies (Gaunt et al., 2010). Accurate identification of co-infections is thus imperative to have a definite diagnosis, to formulate appropriate treatment protocol accordingly, for effective management.

Examining risk factors, the significant association of *B. gibsoni* prevalence in tick exposed dogs in Khordha district may relate to the documented abundance of *Rhipicephalus* and *Haemaphysalis* tick vectors in these localities (Sahu et al., 2013), though further entomological and ecological studies are warranted.

The climate of Odisha is characterized by three main seasons namely summer (March-June), rainy (July- October) and winter (November- February) having variable ambient air temperature (19 °C −31 °C), 76.9% average relative humidity and average rainfall of 1400–1500 mm annually with abundance of ticks in summer and rainy season (Sahu et al., 2013). Climatic conditions favoring tick proliferation during summer and rainy season likely underpin the seasonal peaks in *, B. gibsoni, H. canis* and *A. platys* infections, aligning with tick biology and activity patterns in Odisha’s tropical climate. The absence of seasonal influence on *E. canis* and *B. vogeli* prevalence aligns with prior findings (Kaur et al., 2020).

The assessment of the prevalence of these parasitic infections previously revealed non-significant variations with respect to age, sex and breed (Kaur et al., 2020; Manoj et al., 2020), which also stands true in this study except for *B. vogeli* that revealed significant association with younger age group (<1 yr). *Babesia vogeli* is known to be less virulent, causing mild clinical illness, the infection is eventually cleared up by immune-competent animals. However, it may lead to the development of severe and potentially fatal hemolytic anemia in puppies (Salem & Farag, 2014).

The absence of ticks, regular anti-tick treatments, and strict in-house confinementwere found to have significant role in minimizing the haemoparasitic infections in dogs (Abd Rani et al., 2011; Do et al., 2024; Selim et al., 2021).**.**As ticks are the putative vector for the diseases, integrated tick control with chemical acaricides plays a crucial role in the disease prevention (Atif et al., 2021). Unexpectedly, dogs with limited outdoor activity showed higher infection rates than those fully unrestricted. This might be due to the fact that outdoor activity of pet dogs near vegetation during dusk and dawn increases the chance of tick exposure (Noden et al., 2007). Although feral dogs have greater exposure to tick vectors and more frequent contact, their immune system appears to be better adapted to haemoparasitic infections than that of owned dogs and the notably higher expression of the granzyme B gene in stray dogs, compared with owned dogs, contributes to the commencement of apoptotic pathways within their immune defense (Temizkan & Sonmez, 2022). *B. vogeli* was found to be unaffected by these three factors, which might be associated with its lower prevalence and size of canine population sampled. However, data on prior antiprotozoal and antibiotic treatments in dogs, excluded from our study, are warranted because empirical administration of drugs (such as doxycycline) for minor ailments like pyrexia or as chemoprophylaxis, particularly during tick season often act as contributing risk factor to the prevalence of TBP infections and associated disease states. Moreover, in this study, dogs were sampled from a clinical setting based on a history or the presence of tick infestation, which may introduce selection bias. This limitation could be addressed in future studies through routine random sampling across the region. The prevalence of clinical affection encountered herein in *H. canis, B. vogeli, E. canis,* appeared to be subtler or less profound in this limited number and aligns with previous reports (Bouzouraa et al., 2016; Mittal et al., 2017; Mondal et al., 2021). On the contrary, the higher prevalence of clinical manifestations of *B. gibsoni* infection observed herein supports the notion of existence of wide range of manifestations from asymptomatic form to complicated form of disease with additional organ failure (Mittal et al., 2019). Concomitant *E.canis* and *B. gibsoni* infection reported to cause additive potentiation of pathogenicity and intensify the disease severity (Mittal et al., 2017). *A. platys* co-infections also resulted in enhanced prevalence of clinically affected dogs compared to *A. platys* alone in contrast to previous report that revealed no difference regarding the relative frequencies of clinical and laboratory findings (Bouzouraa et al., 2016). Likewise, triple infections showed clinical signs in 100% affected dogs, suggesting additive pathogenicity.

The Odisha isolates across all five pathogens (*H. canis, B. gibsoni, B. vogeli, E. canis, and A. platys*) demonstrated close phylogenetic relationships with other Indian and global isolates indicating conserved gene sequences within key genetic markers alongside ongoing gene flow (Mittal et al., 2017). The Odisha isolates aligned well within the established genetic boundaries for these species, solidifying the utility of gene markers for epidemiological tracking and comparative studies. Gene flow and mobility of ticks/hosts that has preserved genetic homogeneity might be helpful for implications of regional control strategies.

## 5 Conclusion

In the present investigation, a hexaplex PCR assay was developed and standardized in-house for the simultaneous detection and differentiation of five major canine tick-borne hemoparasites, namely *Babesia gibsoni, Babesia vogeli, Anaplasma platys, Ehrlichia canis and Hepatozoon canis,* with parallel amplification of the canine β-actin gene as an internal control. The assay demonstrated high analytical sensitivity, reliably detecting as little as 0.1–10 pg of parasite DNA without any nonspecific amplification. The epidemiological component relied on tick exposed dogs revealed the highest molecular prevalence of *A. platys* in the study and documented a high prevalence of *B. gibsoni* in Khordha district, with *H. canis, B. gibsoni* occurring more frequently during the summer season and *A. platys* in rainy season. These findings warrant additional investigation into the carrier status of local tick populations to clarify their role in pathogen maintenance and transmission. Co-infections of tick-borne pathogens amplified the disease severity. Phylogenetic analysis indicated relatively stable regional pathogen populations, with no marked genetic divergence or novel genotypes in the examined gene regions, a feature that may be advantageous for long-term diagnostic marker reliability and vaccine design. Nevertheless, confirmation of this apparent evolutionary stability may require extended molecular characterization targeting multiple genetic loci. Overall, the developed hexaplex PCR appears to be a rapid, reliable and highly sensitive tool suitable for high-throughput screening of clinical samples and for large-scale epidemiological surveys aimed at formulating evidence-based control strategies against canine tick-borne infections.

## Funding sources

This research work did not receive any specific grant from funding agencies in public, commercial, or not-for-profit sectors. The work was accomplished by routine institute support.

## Ethical approval

The blood sampling was carried out by registered professionals following the guidelines stipulated by Committee for Control and Supervision of Experiments on Animals (CCSEA). Thu use of the dogs in the protocol was approved by Institutional Animal Ethical Committee (IAEC).

## Data availability

The data supporting the results presented in the article can be found in the tables, figures and supplementary files presented in the manuscript.

## Statement for studies on human animal

The author(s) state(s) that the research entitled “Development of hexaplex PCR assay for the detection and characterization common tick-borne pathogens in dogs, and analysis of risk factors” was conducted in accordance to animal protocols approved by the Institutional Animal Ethical Committee Regd. No- 433/CPCSEA, College of Veterinary Science and Animal Husbandry, Odisha University of Agriculture and Technology, Bhubaneswar, India vide reference no 833/IEAC dated 7.10.2023.

## CRediT authorship contribution statement

**Mirashree Pati:** Writing – original draft, Methodology, Investigation, Data curation, Conceptualization. **Ramesh Chandra Patra:** Formal analysis. **Manaswini Dehuri:** Resources, Formal analysis. **Smruti Ranjan Mishra:** Validation, Methodology. **Sangram Biswal:** Writing – review & editing, Supervision, Funding acquisition. **Geeta Rani Jena:** Supervision. **Chinmoy Mishra:** Data curation. **Dillip Kumar Karna:** Formal analysis.

## Declaration of competing interest

This has reference to submission of an original research manuscript entitled “Development of hexaplex PCR assay for the detection and characterization common tick-borne pathogens in dogs, and analysis of risk factors” authored by Mirashree Pati, Ramesh C. Patra, Manaswini Dehuri, Smruti R. Mishra, Sangram Biswal, Geeta R. Jena, Chinmoy Mishra and Dilip Kumar Karna for publication consideration. It is further declared that there are no known competing financial interests or personal relationships that could have appeared to influence the work reported in this manuscript.

## Appendix Supplementary materials

Image, application 1
