# Supplementary material for: Development of hexaplex PCR assay for the detection and characterization common tick-borne pathogens in dogs, and analysis of risk factors
Source: Vet Anim Sci. 2026 May 19;33:100705. doi: 10.1016/j.vas.2026.100705 (PMC13235372; doi:10.1016/j.vas.2026.100705)
Supplement: Supplementary file 1 [file mmc1.docx]

| 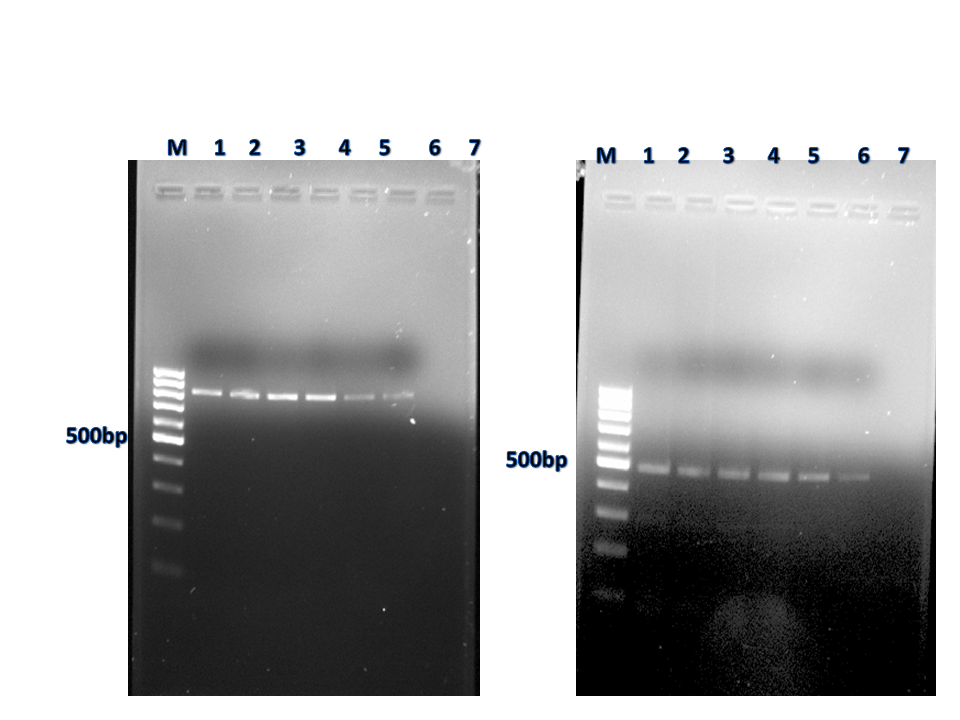 | |
| --- | --- |
| Fig 1A | Fig 1B |
| 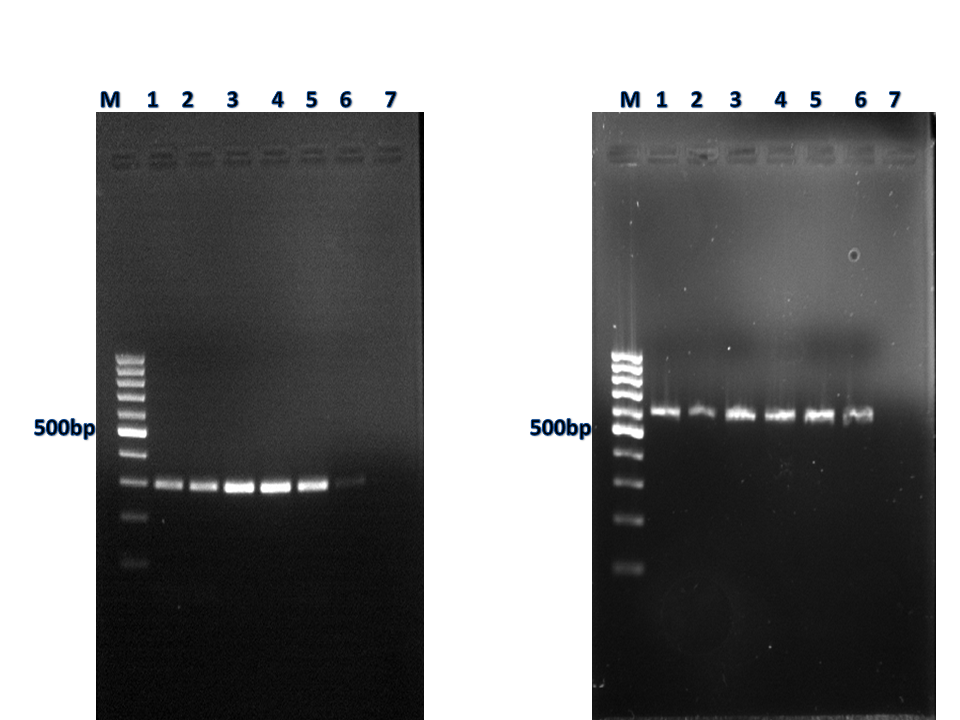 | |
| Fig 1C | Fig 1D |
| 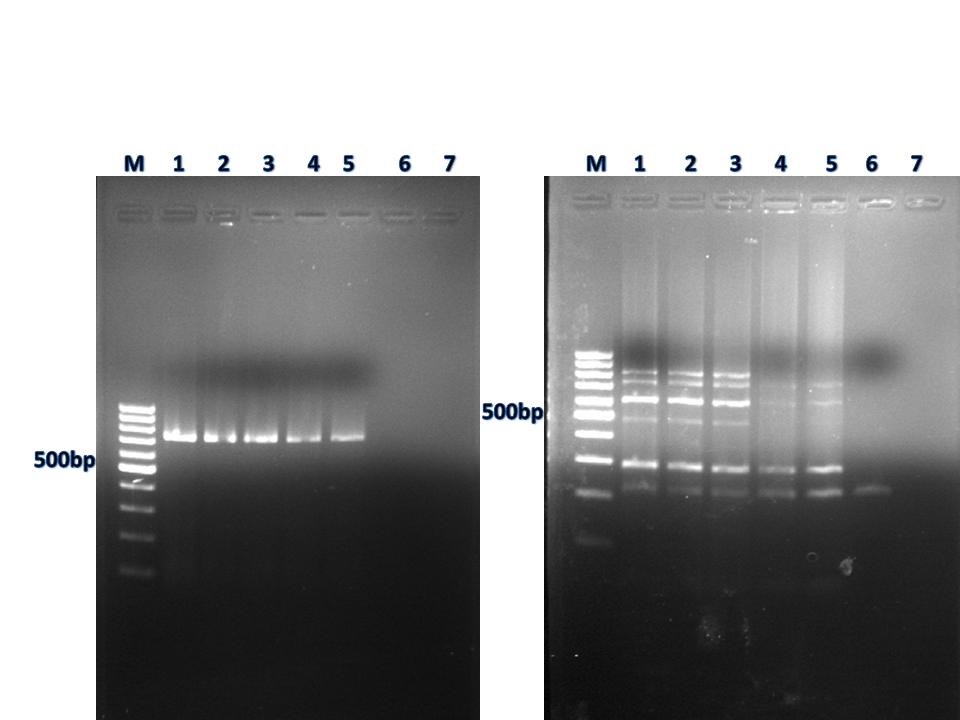 | |
| Fig 1E | Fig 1F |

Fig.1: Singleplex and Hexaplex PCR assays for the detection of Fragments of 817 bp of *Ehrlichia canis* (Fig 1A)*,* 489 bp of *Babesia gibsoni* (Fig. 1B)*,* 292 bp of *Anaplasma platys* (Fig 1C), 602bp of Babesia *vogeli* (Fig 1D), 737 bp of *Hepatozoon canis* (Fig 1E), hexaplex PCR with mixed DNA and canine β-actin gene of 218 bp (Fig 1F). (M: Gene Ruler 100 bp DNA ladder (Thermo Scientific); Lane 1–7: 10-fold serial dilutions of DNA from 1ng/μl to 1 fg/μl)

| 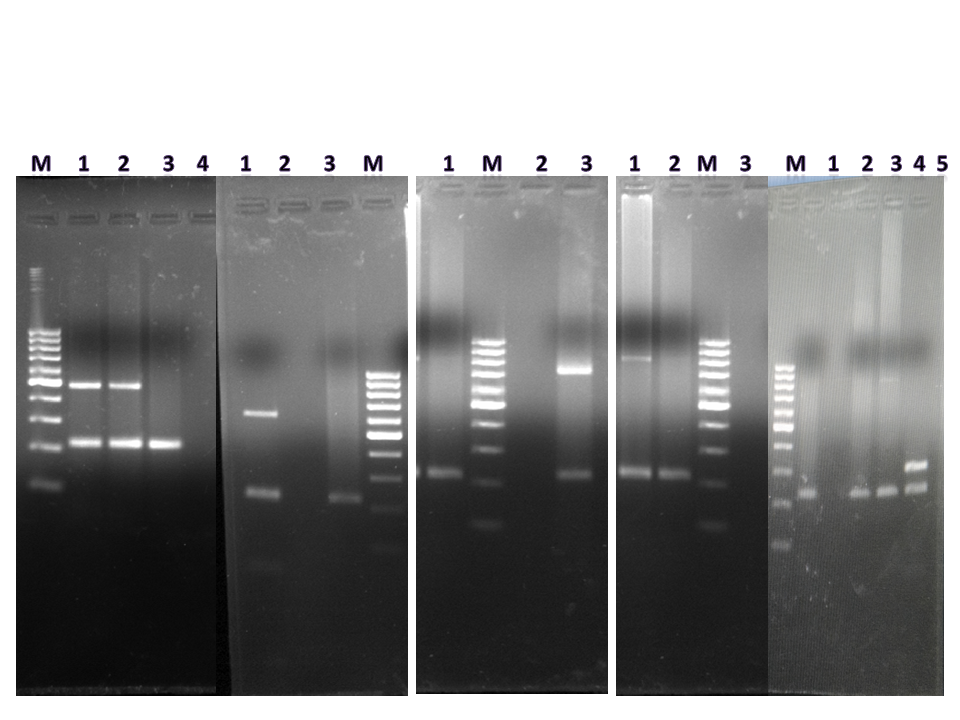 | | | | |
| --- | --- | --- | --- | --- |
| 2A | 2B | 2C | 2D | 2E |

Fig.2: Hexaplex PCR assays of positive control sample. 489 bp of *Babesia gibsoni* along with negative control and Non template control (NTC) (Fig 2A)*,* 602bp of Babesia *vogeli* along with negative control and NTC (Fig. 2B)*,* Fragments of 737 bp of *Hepatozoon canis* along with negative control and NTC (Fig 2C), 817 bp of *Ehrlichia canis* (Fig 2D), 292 bp of *Anaplasma platys* (Fig 2E), (M: Gene Ruler 100 bp DNA ladder (Thermo Scientific)
